# Clinical and epidemiological profile of mycetoma patients from a tertiary care center in Karachi, Pakistan^☆^^☆☆^

**DOI:** 10.1016/j.abd.2020.08.024

**Published:** 2021-07-14

**Authors:** Yousuf Abd Mallick, Nausheen Yaqoob

**Affiliations:** The Indus Hospital, Karachi, Pakistan

Dear Editor,

Mycetoma is a chronic, suppurative and granulomatous infection of soft tissues and bones, which can involve any body part, but feet are the site of predilection. The famous triad consists of the formation of multiple draining sinuses, the presence of colored grains and painless subcutaneous swellings.^1^ Bacteria and fungi both are responsible for this condition, causing “actinomycetoma” and “eumycetoma” respectively. Mycetoma is predominantly reported from a typical geographical zone, known as “mycetoma belt”, which exists between 15° South and 30° North over the globe and has an abundance of different plant species, especially Acacia, with long, sharp, and firm thorns. These thorns, in turn, promote the deposition of spores deep into the tissues upon penetrating injuries.^2^, ^3^

Mycetoma has no documented zoonotic transmission, neither vector nor any animal reservoir.^4^ Rural areas are most affected and those who work barefoot are predominant victims like farmers, shepherds, dairy farm workers, gardeners, livestock workers, and daily laborers. No age is immune to mycetoma but it has been seen more often in young males between 20 to 40 years of age with a male to female ratio of 3:1.^4^, ^5^

This was a retrospective, descriptive study conducted at a tertiary care center in Karachi, a city in southern Pakistan. Untreated or inadequately treated mycetoma cases of all age groups and gender, visited the institution’s dermatology outpatient department between October 2017 to March 2020 were selected. Patients who had completed treatment from other hospitals, amputated cases, and those having concomitant tuberculosis, Human Immunodeficiency Virus and Acquired Immunodeficiency Syndrome were excluded.

Two or more biopsies were done in each patient and sent for histopathology and tissue culture. Extraction of grains, followed by potassium hydroxide mount, Gram’s staining, acid-fast staining, and extensive microbiological cultures were performed in all cases.

X-rays and Magnetic Resonance Imaging (MRI) with contrast was done as a baseline investigations to detect the extent of disease and bone involvement. Bone involvement on MRI was labeled as none, minimal (1 to 3 bones involved) and extensive (more than 3 bones involved).

Permission from the institutional ethical review committee was taken. Study IRB Number: IRD_IRB_2020_04_004.

In this study, out of 12 selected patients; 7 were male and 5 were female with M:F ratio of 1.4:1. Only one patient was a child, mean age of adults was 43.54 ± 11.76 years. Nine patients belonged to rural areas with a Rural to Urban ratio of 3:1. The most common profession of affected patients was farmers (5 patients), followed by daily laborers (2 patients), housewives (2 patients), shopkeepers (1 patient), and student (1 patient). The foot was affected in 10 patients while the hand and knee were affected in 1 patient each. The mean duration of delay in diagnosis was 7 years (Table 1).

**Table 1:** Socio-demographic details of mycetoma cases.

Case nº	Age (years)	Sex	Residence	Occupation	Body region	Delay in diagnosis
1	44	F	Urban	Housewife	Right foot	14 years
2	60	M	Rural	Shopkeeper	Right foot	3 months
3	45	M	Rural	Fisherman	Right hand	12 years
4	48	M	Rural	Farmer	Right foot	8 years
5	64	F	Rural	Farmer	Right foot	10 years
6	41	M	Rural	Farmer	Left foot	12 years
7	49	M	Rural	Farmer	Left foot	7 years
8	9.3	F	Rural	Student	Left foot	3.5 years
9	32	F	Urban	Housewife	Right foot	3 years
10	40	M	Rural	Daily laborer	Left foot	7 years
11	26	M	Urban	Daily laborer	Left foot	3 years
12	30	F	Rural	Farmer	Right knee	6 years

Histopathology helped in 9 cases to establish a diagnosis. Six and 2 biopsies were in favor of eumycetoma and actinomycetoma respectively, while 1 showed features of both organisms i.e., a mixed infection with actinomycetes and fungi which was later confirmed by cultures (Fig. 1).

**Figure 1:**
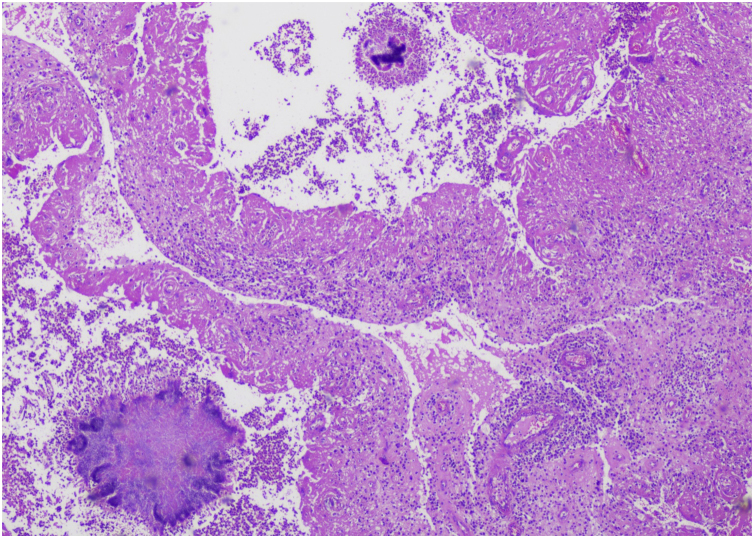
Histopathology showing two different types of colonies (Hematoxylin & eosin, ×10).

The culture was positive in 9 cases and reported fungal, bacterial, and mixed organisms in 5, 3, and 1 case, respectively. Cultures remained negative in 3 cases despite every effort to grow on different aerobic, anaerobic, microaerophilic, and Modified Sabouraud agar media. Aspergillus was reported as the most common species in 5 patients. The rest of the reported organisms and the color of grains are mentioned in Table 2.

**Table 2:** Histopathological and microbiological details of mycetoma cases.

Case nº	Colour of grains	Biopsy findings	Culture	Type of species	Name of species	Final diagnosis	Bone involvement
1	White	M.M.	Positive	Both	Actinomadura madurae & Aspergillus fumigatus	Mixed mycetoma	Minimal
2	White	I.C.	Positive	Bacteria	Streptomyces species	Actinomycetoma	None
3	Black	F.M.	Positive	Fungi	Madurella mycetomatis	Eumycetoma	Extensive
4	Black	F.M.	Positive	Fungi	Aspergillus niger	Eumycetoma	Minimal
5	White	B.M.	Positive	Bacteria	Actinomadura madurae	Actinomycetoma	Extensive
6	Black	F.M.	Positive	Fungi	Aspergillus niger	Eumycetoma	None
7	White	F.M.	Positive	Fungi	Aspergillus flavus	Eumycetoma	Minimal
8	Red	B.M.	Positive	Bacteria	Actinomadura pelletieri	Actinomycetoma	Extensive
9	Black	F.M.	Negative	None	None	Eumycetoma	Extensive
10	Yellow	F.M.	Negative	None	None	Eumycetoma	Extensive
11	Yellow	I.C.	Negative	None	None	None	Extensive
12	White	I.C.	Positive	Fungi	Aspergillus flavus	Eumycetoma	None

B.M., Bacterial Mycetoma; F.M., Fungal Mycetoma; I.C., Inconclusive; M.M., Mixed Mycetoma.

The most striking feature reported in this study was the detection of *Aspergillus niger* in 2 patients, which is a novel fungal species associated with mycetoma (Fig. 2). Both patients were residents of the Eastern part of province Sindh, a farmer in wheat fields, and lived in similar environmental conditions. Despite being a late diagnosis, years after acquiring the infection, there was none to minimal bone involvement in them.

**Figure 2:**
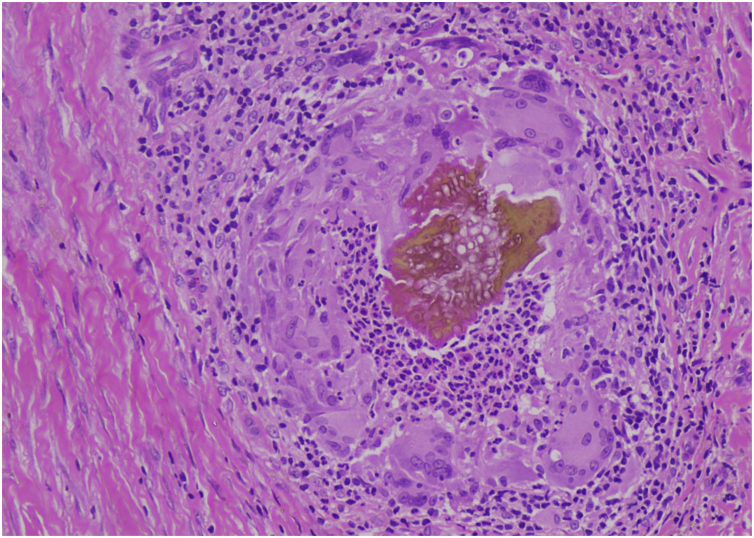
Septate fungal hyphae with hyaline budding surrounded by multinucleated giant cells, neutrophils, eosinophils, and lymphocytes (Hematoxylin & eosin, ×40).

A final diagnosis was established in 11 patients (91.67%). Out of these, 7 were eumycetoma, 3 were actinomycetoma and 1 was mixed infection while 1 patient still remains undiagnosed. The ratio of eumycetoma to actinomycetoma was 2.3:1 (7 vs. 3).

Bone involvement was variable and did not correlate with the species involved or final diagnosis (Table 2). Only one patient (case # 12) had metastatic deposit in the right inguinal region from the primary locus at the right knee. The culture showed *Aspergillus flavus* species from both sites.

Voriconazole 400 mg/day and terbinafine 500 mg/day were given in 6 and 1 patients of eumycetoma respectively for at least 6 months. Response to therapy was poor and amputation was planned in 3 patients. Others responded variably on voriconazole and surgical debulking was required in the majority.

Both patients with *Aspergillus niger* eumycetoma received voriconazole for 1 year and achieved complete clinical and microbiological cure, one with voriconazole alone and the other with associated surgical management, with no relapse of disease on MRI on follow-ups.

Among actinomycetoma cases, 1 case with Streptomyces species mycetoma was treated with a combination of co-trimoxazole and rifampicin with excellent response and disease clearance after 6 months. The other 2 cases with Actinomadura species were treated with Welsh regimen (Amikacin and Co-trimoxazole combination) with partial response and needed surgical debulking later on.

A mixed mycetoma case was treated with antibiotics (amoxycillin 1000 mg/day for 18 months along with linezolid 1200 mg/day for 6 months) and antifungals (itraconazole 400 mg/day for 6 months followed by voriconazole 400 mg/day for 15 months). Sequestrectomy was done once. MRI scans on follow-up did not reveal any signs of disease.

In conclusion, mycetoma is a potentially preventable disease. Protective measures, patient education with early diagnosis and treatment can save many patients from permanent disabilities and amputations. This study showed eumycetoma is more common in the southern part of Pakistan and Aspergillus is the predominant species responsible for this. A novel fungal agent *Aspergillus niger* is also prevalent in this part of the World and responsible for causing mycetoma in immunocompetent individuals.

## Financial support

None declared.

## Authors’ contributions

Yousuf Abd Mallick: Conception and design of the study; critical review of the literature; elaboration and writing of the manuscript; approval of the final version of the manuscript.

Nausheen Yaqoob: Conception and design of the study; collection and analysis of data; drafting of the article; critical review of the manuscript; approval of the final version of the manuscript.

## Conflicts of interest

None declared.
